# A survey on missing data in machine learning

**DOI:** 10.1186/s40537-021-00516-9

**Published:** 2021-10-27

**Authors:** Tlamelo Emmanuel, Thabiso Maupong, Dimane Mpoeleng, Thabo Semong, Banyatsang Mphago, Oteng Tabona

**Affiliations:** Department of Computer Science and Information Systems, Botswana International University of Science and Technology, Palapye, Botswana

**Keywords:** Missing data, Imputation, Machine learning

## Abstract

Machine learning has been the corner stone in analysing and extracting information from data and often a problem of missing values is encountered. Missing values occur because of various factors like missing completely at random, missing at random or missing not at random. All these may result from system malfunction during data collection or human error during data pre-processing. Nevertheless, it is important to deal with missing values before analysing data since ignoring or omitting missing values may result in biased or misinformed analysis. In literature there have been several proposals for handling missing values. In this paper, we aggregate some of the literature on missing data particularly focusing on machine learning techniques. We also give insight on how the machine learning approaches work by highlighting the key features of missing values imputation techniques, how they perform, their limitations and the kind of data they are most suitable for. We propose and evaluate two methods, the k nearest neighbor and an iterative imputation method (missForest) based on the random forest algorithm. Evaluation is performed on the Iris and novel power plant fan data with induced missing values at missingness rate of 5% to 20%. We show that both missForest and the k nearest neighbor can successfully handle missing values and offer some possible future research direction.

## Introduction

Missing values are usually attributed to: human error when processing data, machine error due to the malfunctioning of equipment, respondents refusal to answer certain questions, drop-out in studies and merging unrelated data [1, 2]. The missing values problem is usually common in all domains that deal with data and causes different issues like performance degradation, data analysis problems and biased outcomes lead by the differences in missing and complete values [3]. Moreover, the seriousness of missing values depend in part on how much data is missing, the pattern of missing data, and the mechanism underlying the missingness of the data [4]. Missing values can be handled by certain techniques including, deletion of instances and replacement with potential or estimated values [5–7], a technique denoted as imputation [8]. Several traditional statistical and machine learning imputation techniques such as mean, regression, K nearest neighbor, ensemble based etc, have been proposed in the literature to handle missing values [9, 10]. In some cases, hybrid approaches [11–15], have also been utilized to solve the weaknesses of the traditional imputation techniques. However, it is important to note that the only suitable solution comes down to a virtuous design and good analysis [16]. This is because analysis of performance is dependent but not limited to several factors such as the type of algorithm selected, attribute selection and sampling techniques. Also, as the era of big data is here, data has become large and complex that it is difficult to deal with missing data using traditional learning methods since the established process of learning from conventional methods was not designed to with big data [17]. Therefore, when dealing with missing data, approach is always crucial since improper handling may lead to drawing inaccurate inferences.

In this study, we discuss missing values in “Missing data patterns and mechanisms” section, where we also introduce missing data patterns and mechanisms. “Missing values approaches” section empirically discusses approaches in literature for handling missing values and critically review several implementations in different domains, mostly focusing more on machine learning. In “Performance metrics for missing data imputation” section, we discuss several performance metrics in the missing values domain and “Comparisons” section discusses and analyse results from previous works. We then implement two machine learning algorithms using the Iris data-set on “Experimental evaluation on machine learning methods” section and discussed the results. Finally, “Conclusion and future work” section summarises the paper and point out potential directions for future exploration.

## Missing data patterns and mechanisms

In this section, we discuss the missing patterns in data and different missing data mechanisms.

### Missing data patterns

**Fig. 1:**
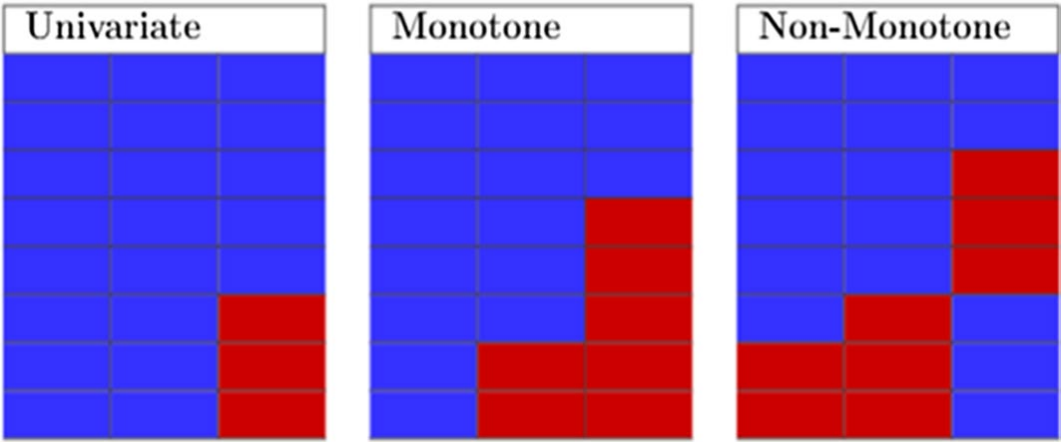
Representation of missing data patterns data. Blue represents observed values; red is missing values

Missing data patterns describe which values are missing and observed in a data set. However, there is no standard list of missing data patterns in the literature as discussed in [18–20]. In this subsection, we discuss three missing data patterns that appear most in the literature which are univariate, monotone and non-monotone. In Fig. 1 we further demonstrates the different patterns in missing data.

Univariate: Missing data pattern is univariate when there is only one variable with missing data [21]. This pattern is rare in most disciplines and arises in experimental studies [22].

Monotone: Missing data pattern is said to be monotone if the variables in the data can be arranged, this pattern is usually associated with a longitudinal studies where members drop out and never return [23]. The monotone data pattern is easier to deal with since patterns among the missing values are easily observable [24].

Non monotone: This is a missing data pattern whereby the missingness of one variable does not affect the missingness of any other variables [25].

### Missing data mechanisms

Mostly mechanisms that lead to the missing values on data affect some assumptions supporting most missing data handling methods, hence, in the literature the missing data has been defined according to these mechanisms. The authors of Rubin [26] established the missing data theory, categorized by three main mechanisms for missingness, which are defined depending on the available and missing data. To define missingness, let *Y* be a matrix of the entire data set that is decomposed into $Y_o$ and $Y_m$, which denote the observed and missing data. Let *R* denote a missing value matrix defined by,

$$R := \left\{ \begin{array}{ll} 0, \quad \text{if} \; \text{Y} \; \text{is} \; \text{observed}\\ 1, \quad \text{if} \; \text{Y} \; \text{is} \; \text{missing} \end{array}\right.$$

Let *q* represent a vector of values that indicate the association between missingness in *R* and the data set *Y*. The missing values mechanisms are therefore defined by the probability of whether a value is observed or missing as we outline below.

#### Missing completely at random (MCAR)

This is when missing observations are not reliant on the observed and unobserved measurements. The probability of MCAR is defined as:

1 $$p( R|q )$$

#### Missing at random (MAR)

The likelihood of a missing value in MAR is only related to the observable data. The probability for MAR can be defined as:

2 $$p(R | Y_o,q )$$

Missing at random (MAR) is mostly encountered in health science studies data sets. Under this mechanism, missing values can be handled by observed predictor variables [27].

#### Missing not at random (MNAR)

This refers to when missing data is neither MCAR nor MAR. The missing data depends equally on the missing and observed values. In this method, handling the missing values is usually impossible, as it depends on the unseen data. The MNAR probability is defined as:

3 $$p(R |Y_o, Y_m,q )$$

The probability of whether a position *R* is missing or observed depends on both $Y_o$ and $Y_m$. This mechanism is mostly applied in different domains predominantly in the domain of (bio)medicine [28], but is also applied in the psychological and educational data-sets [29, 30].

According to Graham and Dantan et al. [9, 31], it is mostly impossible to unambiguously categorise missing data into these three mechanisms since imagining that missing data is completely not related to other non missing variables is very challenging because one way or the other missing values relate to non-missing variables. Many researchers, however, report that the easiest way is to complete all the missing data as MAR to some degree because MAR resides in the middle of this continuum [9].

## Missing values approaches

In this section we discuss missing values approaches available in the literature. We also review implementation of missing values approaches in various domains.

### Deletion

In this approach all entries with missing values are removed/discarded when doing analysis. Deletion is considered the simplest approach as there is no need to try and estimate value. However, the authors of Little and Rubin [18] have demonstrated some of the weakness of deletion, as it introduce bias in analysis, especially when the missing data is not randomly distributed. The process of deletion can be carried out in two ways, pairwise or list-wise deletion [32].

#### List-wise or case deletion

In list-wise deletion, every case that has one or more missing values is removed. List-wise deletion has become the default choice when analysing data in most statistical software packages [33]. However, under the assumption that the data is not MCAR, list-wise results in biasness [34]. While, if the data samples are large enough and the MCAR assumption is satisfied, then list-wise deletion may be a reasonable approach. If the sampled data is not large, or the MCAR assumption is not satisfied, then list-wise deletion is not the best approach to consider. List-wise deletion may also result in losing some important information, especially when the discarded cases are high in numbers.

#### Pairwise deletion

To mitigate against information loss when doing do list-wise deletion one can use pairwise deletion. This is because pairwise deletion is carried out such that it reduces losses that could occur in list-wise deletion. This is done by eliminating values only when there is a certain data point needed to test if the value assumed to be missing is in fact missing [35]. The weakness of pairwise deletion is that it can lead to an inter-correlation matrix that is not positive definite, which is can possibly prevent further analysis such as calculating coefficients estimates [36]. Finally, pairwise deletion also known to produce low bias results for MCAR or MAR data [34].

### Imputation

The process of imputation involves replacing missing values by some predicted values. The non-missing values data set is normally used to predict the values used to replace the missing values [8]. In the following we cover some of the most used imputation methods in the literature.

#### Simple imputation

Simple imputation approach entails replacing missing values for each individual value by using a quantitative attribute or qualitative attribute of all the non-missing values [37]. With simple imputation, missing data is handled by different methods such as, mode, mean, or median of the available values. In most studies simple imputation methods are used because of their simplicity and that they can be used as an easy reference technique [38]. However, simple imputation methods may produce bias or unrealistic results on a high-dimensional data sets. Also, with the generation of big data emerging, this method seems to be performing poorly and therefore is inadequate to be implemented on such data sets [39].

#### Regression imputation

Regression is one of the preferred statistical technique for handling missing values. This method is also termed conditional mean imputation, here missing values are replaced with a predicted value created on a regression model if data is missing at random. The overall regression process is a two-phase method: the first step, uses all the complete observations to build a regression model, and imputes missing data based on the built regression model [40]. The regression method is decent since it maintains the sample size by preserving all the observations with missing values. However, regression may need a large sample of data to produce stable results. Furthermore, a single regression curve is followed for all the imputed values and no inherent variation is presented in the data [18]. Considering a feature containing missing values, and the remaining attributes are complete. A regression model approximates the missing features using the available data. The first step is to estimate a set of regression equations that will predict the incomplete values from the complete values using a complete case. Predicted values are then generated for the incomplete variables. These predicted values fill in the missing values. For the imputation of *y* variables given a set of variables $j_{1},\ldots,j{q}$, a regression model is used as follows:

4 $$y= \alpha +\beta _{1}j_{1}+\cdots+\beta _{q}j_{q}+\epsilon$$

with $\alpha$, $\beta _{1},\ldots,$ $\beta _{q}$ being the unknown values and $\epsilon$ is a distance variable. The estimates in Eq. 4 will results in a prediction for *y* given by the variables:

5 $${\hat{y}}=a+b_{1}j_{1}+\cdots+b_{q}j_{q}$$

with *a* $b_{1}$ $b_{q}$ denoting the least squares estimates of $\alpha$, $\beta _{1},\ldots,$ $\beta _{q}$. An imputation ${\tilde{y}}$ is then made

6 $${\tilde{y}}= {\hat{y}}=a+b_{1}{j_{1}}_{i}+\cdots+b_{q}{j_{q}}_i$$

The technique of regression implemented depend on the nature of the data. If there are two or more missing features, a multivariate regression model must be used for imputation [41]. Multivariate Regression measures the degree at which more than one independent prediction and more than one dependent responses, are linearly related [42]. A multivariate regression imputation is used as follows using the extension of a standard regression model in Eq. 4:

7 $$y=\mu _{y}+B_{yj}(j-\mu _{j})+\epsilon$$

where the target value in *y* is retrieved by using the same vector of variables *j*. An expectation maximization algorithm is then used to find the estimates of the parameters in 7, the algorithm uses the information of the observed data to estimate the parameters. More information on the expected maximisation is presented on De Waal et al. [43]. After obtaining estimates of the unknown parameters in Eq. 7, the imputation of missing values in *y* is obtained as before from the observed vector $j_i$. Then an imputation is retrieved directly from the predicted value,

8 $$\tilde{y_{i}}=\hat{y_{i}}={\hat{\mu }}_{y}+\hat{B_{yj}}(j_{i}-{\hat{\mu }}_{j})$$

and an imputation is done by adding a random disturbance to the prediction:

9 $$\tilde{y_{i}}=\hat{y_{i}}+e_{i}={\hat{\mu }}_{y}+\hat{B_{yj}}(j_{i}-{\hat{\mu }}_{j})+e_{i}$$

A common choice is to get $e_i$ from a multivariate distribution with a mean vector zero and the residual of the regressions *y* on *j* [43].

In research studies using the regression approach includes one by Sherwood et al. [44], where a weighted quantile regression approach that estimated missing values in health data was conducted. The authors used a quantile regression approach on the health data because it is usually attributed to a high level of skewness, heteroscedastic variances and the weighted quantile regression estimator is consistent, unlike the naive estimator, and asymptotically normal making it suitable for analysing this type of data. The experiment demonstrated the effectiveness of the quantile regression technique on the numeric health care cost data analysis. However, the estimator used fully observed observations and was most suitable when the rate of the missing data was not excessively high. Moreover, the approach was not robust due to functional form specification and could have introduced bias results.

In another study, the authors proposed a complete case regression missing values handling method using functional principal component [45]. The performance of the approach when the missing values were not handled was experimented on and compared with regression imputed missing values. Their major interest in the study was the functional linear regression when some observations of the actual response were missing.

Another study applied a multivariate imputation technique for imputing missing values in normal multivariate data. The imputation values were obtained from the sequence of regression, where all the variables containing missing values were regressed against the variables that did not contain missing values as predictor variables by using the iteration approach. The approach worked well with more one variable containing missing values and non-monotonous patterns [46].

#### Hot-deck imputation

Hot-deck imputation handles missing values by matching the missing values with other values in the data set on several other key variables that have complete values [47]. The method has variations, but one that allows natural variability in missing data selects a pool of all cases. This pool is called the donor pool, that is identical to the cases with missing data on many variables and chooses one case randomly out of that pool. The missing value is then replaced by data from the randomly chosen cases. Another technique involves replacing the closest donor neighbor rather than selecting one donor from a pool of donors [48]. However, the method disregards the variability in missing data. The other variations of this imputation technique are weighted random hot-deck and weighted sequential hot deck. The weighted random hot deck method does not limit the number of times a donor is nominated; however, the donors are chosen randomly from the donor pool. In contrast, weighted sequential hot-deck puts a restriction on the amount of time a donor can be chosen to prevent the same donor to be paired with a large quantity of recipients [47].

The hot-deck method is very popular in all single imputation methods as it results in a rectangular data [47], that can be used by secondary data analysts. Also, the method avoids cross-user inconsistency and does not depend on model fitting for the missing value to be replaced, making it possibly less delicate to model specification as compared to a method built on a parametric model, for instance regression imputation. The method also decreases bias in non-response. Even though the method is being used widely in research, its concept is not as well established compared to other imputation techniques.

In Sullivan and Andridge [49], a hot deck imputation method that allowed for the investigation of the impact of missingness mechanisms, ranging from MAR to MNAR, and used the information contained in fully observed covariates was proposed. Bias and coverage of estimates from the proposed technique were investigated by simulation. Results also, showed that the method performed best when fully observed values were associated with the outcome.

In another study Christopher et al. [50], a fractional hot deck imputation method was used to handle missing values. The procedure was applied to the MAR mechanism, but the missing data pattern and the comparison was done with list-wise deletion, mean, median imputation methods only. Their method produced a smaller standard error compared to other method they used for comparison. However, the experiment may have been bias since it was concluded that it performed better being compared to the imputation method that usually produce biased results.

#### Expectation–maximization

The expectation maximization technique is an iterative method for handling missing values in numerical datasets, the algorithm uses an “*impute, estimate and iterate until convergence*” approach. Every iteration includes two stages which are: expectation and maximisation. Expectation estimates missing values given observed data, whereas in maximisation, the present estimated values are used to maximize the probability of all the data [51].

Approaches in research have been proposed to deal with missing values using expectation minimisation. In Rubin et al. [52], an investigation on handling missing data was done using a dataset that analysed the impacts of feeding behaviours among drug-treated and untreated animals. The expectation maximisation algorithm was used and compared to other methods like list-wise deletion which was the least efficacious method, Bayesian approach and the mean substitute regression. The authors concluded that that the EM algorithm was the best method for the type of data they used. However, using real datasets in the study may have led to the results being specific to idiosyncrasies in the dataset and in sampling or are reflective of hypothetical expectations.

In another research, an expected maximisation algorithm was used for imputation to solve the problem of training Gaussian mixtures in large high-dimensional datasets settings with missing values [53]. The imputed datasets were then experimented in classification models and proved to provide a significant performance improvement over other basic missing value imputation methods. However, the expected maximisation technique resulted in expensive matrix computations.

Generally, single imputation methods as discussed above are simple methods to handle missing data and save time. However, these methods are mostly bias, and error of their imputations is not incorporated. Furthermore, single imputation techniques do not represent the vulnerability associated with the missing values [9]. Therefore, researchers have experimented on improved methods to handle missing data that give much better performance [10]. The improved techniques are believed to be unrivalled to the single missing data techniques since they proved to yield unbiased analysis.

#### Multiple imputation

It is evident that missing data handling goes beyond deleting or discarding missing data [26] and therefore researchers resort to multiple imputation. Multiple imputation is where the distribution of the observed data is utilized to approximate numerous values that reflect the uncertainty around the true value, and this method was mostly implemented to solve the limitations of single imputation [54]. The analysis is done on a data set using the various missing data techniques, and the average of parameter estimates across *M* samples is computed into a single point estimate. Thus, multiple imputation technique comprises of three distinct phases:

- Missing data is handled in *M* resulting in *M* complete data sets.
- The *M* complete data sets are then analysed.
- The results of all the *M* imputed data sets are combined for the final imputation result.

Though multiple imputation is set up as a standard methodology for dealing with missing values, it is important for researchers to utilize appropriate techniques for imputation, to guarantee that dependable results are obtained when experimenting with this approach [55]. Furthermore, performance may be affected negatively when carrying out imputation on real data such as survey data, clinical data and industrial data which may be characterized by a high rate of missingness and a great number of factors that are not necessarily linearly related. Also, traditional multiple imputation methods seem to perform poorly on high dimensional data and researchers have resorted to improving these algorithms to enhance their performance [56, 57]. Similarly, there is also evidence that caution should be made for continuous-based techniques when imputing categorical data as this may lead to biased results [58].

We discuss the approaches on the literature on multiple imputation: the researchers in Horton et al. [58], experimented on a technique that accurately imputed missing values on a patient data set using multiple imputation using Least Squares Support Vector Machine (LSSVM). Five datasets were used to determine the performance of the proposed method. The evaluation results illustrated that their method outperformed conventional imputation methods and that the study was a more robust technique that generated values closer to the one that was missing. Moreover, the author also proposed another method Clustered Z-score Least Square Support Vector Machine (CZLSSVM) and demonstrated its efficiency in two classification problems for incomplete data. Their experimental results also indicated that the accuracy of the classification was increased with CZLSSVM, and that the algorithm outperformed other data imputation approaches like SVM, decision tree, KNN, rough sets and artificial neural networks. In another study de Goeij et al. [59], the authors also proposed a multiple imputation method for clinical practice data. The results of the method gave unbiased estimates and standard errors, on MCAR or MAR missing mechanisms. Also, the prediction model specification was adequate, though it may have required the help of a statistician. However, their multiple imputation technique performed better than the other conventional methods. There has been a study also by Khan and Hoque [39], that explored a multiple imputation approach that extended multivariate imputation by chained equation for big data. The approach had presented two variants one for categorical and the other numeric data and implemented twelve existing algorithms for performance comparison. The experimental results of the experiment with four datasets demonstrated that the method performed better for the imputation of binary and numeric data.

#### Imputation methods inspired by machine learning

Imputation methods built on machine learning are sophisticated techniques that mostly involve developing a predictive approach to handle missing values using unsupervised or supervised learning. As other imputation methods these techniques estimate the missing data estimation depending on the information available from the non-missing values in the data using labelled or unlabelled data. Mostly if the available data has useful information for handling the missing values, an imputation high predictive precision can be maintained. We discuss some of the most researched on machine learning imputation techniques below.

#### K nearest neighbour classification

The KNN algorithm works by classifying the nearest neighbours of missing values and use those neighbours for imputation using a distance measure between instances [60]. Several distance measures such as the Minkowski distance, Manhattan Distance, Cosine Distance, Jaccard Distance, Hamming Distance and Euclidean distance can be used for KNN imputation, however the Euclidean distance is reported to give efficiency and productivity [61, 62] and therefore is the most widely used distance measure. We further explain the KNN imputation using the Euclidean distance measure below:

10 $$Dist_{xy}=\sqrt{{\sum _{k=1}^{m}} {(X_{ik}-X_{jk})^{2}}}$$

where $Dist_{xy}$ is the Euclidian distance, *k* is data attributes $j= 1,2,3\ldots k$, *k* data dimensions, $(X_{ik})$: value for $j$− attribute containing missing data and $(X_{jk})$ is the value of $j$− attribute containing complete data.

The value of the k points that have a minimum distance are chosen then Weight Mean Estimation is calculated.

11 $$X_{k}=\frac{{\sum _{j=1}^{J}}w_{j}v_{j}}{\sum _{j=1}^{J}w_{j}}$$

where $X_{k}$ is the mean estimation, *J* is the number of parameters used with j = 1,2,3…*K*. $v_{j}$ are complete values on attributes containing missing data while $w_{j}$ is the nearest neighbors observed. The weighted value is then given by the following equation:

12 $${ w_{j}=\frac{1}{dis_{(x,y)^2}}}$$

The KNN imputation technique is flexible in both discrete and continuous data and can also be implemented as a multiple missing data handler [1, 60]. However, KNN imputation has drawbacks such as low precision when imputing variables and introduces false associations where they do not exist [63]. The other weakness of KNN imputation is that it searches through all the data set, hence increasing computational time [64]. However, there are approaches in literature that have been developed to improve the KNN imputation algorithm for missing values problems, see [65–70].

A KNN imputation using several cases with different mechanisms and missing data models was proposed [71]. The authors concluded that their method performed well in handling missing values. However, the research did not follow any missing value mechanism when manually removing the data for the experiment, which may lead to bias results.

In another research, the authors introduced an iterative KNN imputation method which was an instance-based technique that took advantage of the correlation on the attributes by using grey relational grade as an alternative for Euclidean distance measure to search k-nearest neighbour instances. The imputed data was predicted from these nearest neighbour instances iteratively. This iterative imputation permitted all values from the preceding iteration to be used for missing value estimation. Also, the method was reported to fill in all the missing values with dependable data regardless of the missing rate of the dataset. The experimental results suggested that the proposed method resulted in a better performance than other methods regarding imputation accuracy and convergence speed [72]. However, the dataset that was used here had originally no missing values and the missing values been imputed at random not considering other missing values mechanisms which may have led to unrealistic results.

In another research, a novel grey relational analysis approach for incomplete instances using the KNN imputation technique was experimented on [73]. The approach was experimented on four datasets with different artificial missingness set-ups to investigate the performance of the imputation. The experiential results of the study demonstrated that the approach was superior to traditional KNN imputation. Furthermore, the classification accuracy could be maintained or improved by using this approach in classification tasks.

Another study developed a novel K nearest neighbour (KNN) incomplete-instance based imputation approach called CVBkNN, which utilized cross-validation to improve the parameters for each missing value [74]. Eight different datasets were used for the experiment. The results of the study demonstrated that their approach was superior to other missing values approaches. They also displayed the optimal fixed parameter settings for KNN imputation for software quality data. Their approach proved to improve classification accuracy or at least maintained it. However, determining additional meaningful parameters for configuration could have improved the study’s accuracy further.

In another study by Batista and Monard [75], the KNN algorithm was experimented to evaluate its efficiency as an imputation method to treat missing data and compared its performance to other algorithms such as by the C4.5 and CN2 and the mean or mode imputation method. In the experiment missing values were artificially implanted, in different rates and attributes, into the data sets. The KNN algorithm performed well even in the presence large amount of missing data compared to the other algorithms.

A genetic algorithm enhanced k-nearest neighbour for handling missing values named EvlKNNImpute was also proposed in this study. The KNNImpute has showed effective compared to other methods used in imputation using the yeast dataset [76]. Their approach also proved to perform better when there was an elevated level of missing rate in a data than a small missing rate.

In another study, the authors incorporated correlation matrix for KNN algorithm design. The least-squares loss function was used to minimize the reconstruction error and reconstruct every test data point by using all training data points. Their method, compared with traditional KNN methods, proved to achieve a higher accuracy and efficiency [77]. However, like many other kinds of research in data imputation this study did not consider the influence of missingness mechanisms and patterns on imputation performance.

The KNN imputation method has been highly researched for imputation since it has proved in literature to perform better than other imputation approaches as seen in the reviews above. However, none of the studies systematically analysed the effects of imputation ordering in the KNN imputation performance. Moreover, there is still no proven common resolution to select the optimized KNN parameters for imputation. Although some researchers use different missingness scenarios to evaluate their approaches, the significance of the influences of this missingness mechanisms are often neglected. Also, the use of KNN in the big data setting is still an under-explored area.

#### Support vector machine (SVM)

Another common machine learning algorithm that is extensively used for missing data handling is the SVM [78, 79]. The SVM, for a labelled training sample, efforts to discover an optimal separating hyper-plane such that the distance from the hyper-plane to the nearest data points is maximized [80]. The hyper-planes are defined by

13 $$w\cdot x_1+b \ge +1 \,\ when \,\ y_i=+1$$

14 $$w\cdot x_1+b\le -1 \,\ when \,\ y_i=-1$$

where *w* is a weight vector, *x* is an input vector and *b* is bias.

Like other machine learning algorithms, the imputation of missing values with this method can impact the accuracy and utility of the resulting analysis. Authors of Honghai et al. [78], used the SVM regression-based method for missing data imputation. The experimental procedure set the decision attributes as the condition attribute and the condition attribute as the decision attribute, then SVM regression predicted the condition attribute values. The experimental results proved that the SVM regression approach had the highest precision on the SARS data set. However, the experiment did not report any use of missing value patterns, ratios or mechanisms used. Also, in Smola et al. [81], the authors demonstrated an SVM and Gaussian processes for missing data handling using exponential families in feature space. In this research estimation with missing values become a problem of computing marginal distribution and finding efficient optimization methods. In another approach [82], the authors replaced the missing values by using the results obtained from applying the SVM classifier over the training set and used an SVM regression to handle the values. The authors experimented using the SVM classifier as an imputation approach because it was reported to perform well on text categorisation problems in Joachims [83]. However, the results of the study concluded that the SVM regression approach gave a much better performance compared to the SVM classifier and other classification and regression approaches, though this might have been influenced by the imbalanced dataset used for the experiment. Since imbalanced data may contribute to the increase of performance of SVM regression.

In Chechik et al. [84], handled missing values by max-margin learning framework. They formulated an objective function, which used geometric interpretation of the margin, that aimed to maximize the margin of every sample in its own relevant subspace. They also showed two approaches for optimizing the general case: an estimation that can be solved as a standard quadratic problem and an iterative approach for solving the exact problem. Their methods saved computational time by avoiding the pre-processing step. More importantly, they demonstrated an elegant missing value handling approach which outperformed other methods when the missing values had a significant structure, and the approach also proved to be competitive compared with other techniques when the values are missing at random.

#### Decision tree

The decision tree is a machine learning algorithm that illustrates all conceivable outcomes and the paths leading to those outcomes in the form of a tree structure. Missing values imputation using this method is done by building decision trees to observe the missing values of each variable, and then fills the missing values of each missing variable by using its corresponding tree [85]. The missing values prediction is then shown in the leaf node. Additionally, this algorithm can handle both numerical and categorical variables, identify the most variables and eliminate the rest. However, decision trees can produce a complex tree that tend to be time consuming but have a low bias [86].

Several researchers [82, 87–89] have used decision trees for imputation, and we discuss their input. A decision tree and forest technique for the imputation of categorical and numerical missing values was proposed. The technique identified horizontal segments in the data set where the records belonging to a certain segment had higher similarity and attribute correlations. The missing data were then imputed using the similarity and correlations. Nine real life data sets were used to compare the technique to other existing ones using four regularly used evaluation criteria [87]. Their experimental results indicated a clear superiority of the technique. However, an improvement on their technique for attaining a better computational complexity, and memory usage may be needed.

Also, in a by Gimpy and Rajan Vohra [88], a missing values approach using a decision tree algorithm. A student data set with missing values was used and a classification algorithm was implemented for comparing accuracy with incomplete data and after imputation. As a result, accuracy was higher on imputed data set as compared to incomplete data set. However, in this study there was no report on missingness ratios or mechanisms considered.

In another paper Rahman and Islam [89], the authors presented a missing value handling technique, using decision trees and expectation–maximization algorithm. They argued that the correlations among the attributes in the horizontal partition of a data set could be higher than the correlations over the whole data set. Also, that expectation maximization performance on higher correlations data is expected to be better than on lower correlations data set. Therefore, they applied expected maximization imputation on various horizontal segments of the data with high correlations between the attributes. Also, various patterns of missing values with different missing ratios were used and the experimental results indicated that their approach performed significantly better.

Another study replaced the missing values by applying the Decision Trees approach. The authors pruned the decision tree by learning the pruning confidence over a training set and predicted probabilities keeping the minimum number of instances per leaf to 2. The method was proposed with other methods for handling missing data and the author concluded that the results of different approaches were dataset dependent, and no approach was a solution for all [82].

The three most used decision tree learning algorithms are: *ID3*, *C4.5* and *CART*.

- CART: Classification and Regression Trees (CART) addresses both continuous and categorical values to generate a decision tree and handle missing values. The algorithm identifies a twofold rule based on one indicator variable that segments the data into two nodes by minimizing variance of the outcome within each node. The tree is then developed by proceeding this splitting recursively until reaching a stopping point determined by the tuning parameters. Imputation is then made from a regression tree by identifying the terminal node to which a new subject belongs and sampling from the outcomes in that node [90]. An attribute selection measure Gini Indexing is used in CART to build a decision tree which unlike ID3, C4.5 does not use probabilistic assumptions. Also, CART generates binary splits that produce binary trees which other decision tree methods do not. Furthermore, this method uses cost complexity pruning to remove the unreliable branches from the decision tree to improve accuracy and does not rely upon distributional assumptions on the data [91].
- ID3: This is a decision tree technique that can be built in two stages: tree building and pruning. A top-down, greedy search is applied through a given set to test each attribute at every tree node. Then information gain measure is used to select the splitting attribute. It only accepts categorical attributes when building a tree model and does not give precise outcome when there is noise. Continuous missing values can be handled by this method by discrediting or considering the value for the best split point and taking a threshold on the attribute values. This method does not support pruning by default; however, it can be done after building a data model [91].
- C4.5: This algorithm was developed after the ID3 algorithm and handles both continuous and categorical values when constructing a decision tree. C4.5 addresses continuous attributes by separating the attribute values into two portions based on the selected threshold such that all the values above the threshold is regarded as one child and the remaining as another child. Gain Ratio is used as an attribute selection measure to construct a decision tree. The algorithm handles missing values by selecting an attribute using all instances of a known value for information gain calculation. Instances with non missing attributes are then split as per actual values and instances with missing attribute are split proportionate to the split off known values. A test instance with missing value is then split into branches according to the portions of training examples into all the child nodes [92]. The algorithm withdraws bias information gain when there are many output values of an attribute.

Another popular form of the Decision trees approach is the Random Forest algorithm, which is a stack of decision trees through bagging which combines multiple random predictors to aggregate predictions the prediction rule is based on the majority vote or average over all trees. Forests can achieve competitive or even superior prediction strengths in comparison to well established approaches such as regression and support vector machines [93]. The process of imputing missing values with the random forest includes the following steps as discussed by Breiman [94]:

1. Selecting a random sample of the observations with replacement;
2. A set of variables are then selected at random;
3. A variable providing the best split is chosen;
4. The step of choosing a variable that produces the best split is repeated until the maximum depth is reached;
5. The steps above are repeated until the certain number of trees is reached;
6. A prediction of the missing value is then done upon a majority vote.

There are several studies in literature [95, 96], where Random Forests were used for handling missing values. In a study by Hong and Lynn [97] an extensive simulation study that involved missing at random simulated datasets using random forest imputation and evaluated in comparison with predictive mean matching.

#### Clustering imputation

Clustering methods, such as hierarchical clustering and k-means clustering have been generally experimented for missing data handling in the literature. The K-means clustering technique consists of 2 steps where, in the first step K-means clustering is used to get clusters, then the cluster information is used to handle the missing values [98]. However, clustering methods are reported to not be robust enough to handle the missing data problem. The clustering method can be defined as follows [99]:

Given a data set *T* = ${t_1, t_2,\ldots tp,\ldots T_{N_p}}$ where $T_p$ is a feature vector in the $N_d$-dimensional feature space, this feature vector *t* is a single data point and $N_p$ is the number of patterns in *T*, then the clustering of *T* is the partitioning of *T* into *K* clusters ${C_1, C_2,\ldots,C_K}$ satisfying the following conditions:

- Every feature vector has to be assigned to a cluster
  15 $$\bigcup _{k=1}^{K} {C}_k = {T}$$
- With at least one feature vector assigned to it
  16 $$C_k\ne \phi , k=1,\ldots,K$$
- Each feature vector is assigned to one and only one cluster
  17 $$C_k\bigcap C_{kk} =\phi$$
  where $$k\ne kk.$$

In study by Gajawada and Toshniwal [98], a missing value imputation method was proposed based on K-means clustering. The proposed method was applied to clinical datasets from the UCI Machine Learning Repository. The method proved to perform better than the simple method that did not use imputed values for further imputations. However, errors in earlier imputations may have propagated to further imputations. Hence this point should be considered when applying methods like the proposed method on real world datasets. In another paper, a clustering-based non-parametric kernel-based imputation technique, called Clustering-based Missing value Imputation (CMI), was proposed for dealing with missing values in target attributes [100]. The experimental results demonstrated the algorithm was an effective method in creating inference for variance and distribution functions after clustering. However, the approach did not consider missing values in conditional attributes and class attributes. There has also been advances in imputing big data based on clustering, Besay Montesdeoca et al. [101] proposed a big data k-means clustering, and a big data fuzzy k-means missing values approach that resulted in robust and efficient output for big data and offered reasonable execution times. The two imputation techniques surpassed in most cases mean imputation and elimination of the instances with lost values during classification. Offer robust and efficient results for Big Data datasets, offering reasonable execution times. The fuzzy k-means approach was proved to provide better results for high percentages of missing values in the data, while the k-means performed better with the dataset that had lower amounts of missing values. Zhang et al. [102], also proposed a multiple imputation clustering based approach that handled missing values in big longitudinal trial data in e-Health. The proposed concept proved that it could be easily adapted for different types of clustering for big incomplete longitudinal trial data in eHealth services.

#### Ensemble methods

Ensemble methods are strategies that make multiple models and then combine them to produce a single improved result. This method usually produces more precise results than a single model would. This has been the case in machine learning competitions, where the triumphant models used ensemble techniques [103]. Studies have confirmed that ensemble missing data handling algorithms outperform single base machine learning algorithms [104–108]. Also, ensemble methods can be implemented in parallel computing environments, which are necessary to process missing data in big datasets. These ensemble algorithms are a group of techniques that their decisions are combined in a way to optimize the execution of a specific algorithm [109]. Developing an ensemble involves of certain steps which are creating varied models and merging their estimates (see Ensemble Generation). It is to be noted that ensemble techniques are best suited mostly where the highest possible accuracy is desired [110]. Before an ensemble is created there need to be a strategy in-order to build an ensemble that is as diverse as possible. This is because building the best ensemble method depends much on the problem that is being handled [111]. They are several ensemble strategies that are used, and these include but are not limited to Bagging, Boosting and Stacking.

**Ensemble Generation** The general ensemble algorithm creation which was formalized by [112] consists of two steps as stated above. The steps involve selecting points (creating varied models) and fitting coefficients (merging their estimates) which are explained in detail below.

1. Selecting points $$\left\{ q_m\right\} _{I}^{M}$$
  1. *T*_0_(*x*) = 0
  2. For *m* = 1 to *M*
  3. $${q_m} = {}_q^{argmin}\sum\nolimits_{i \in {S_m}(\eta )} {L\left( {{y_i},{T_{m - 1}}({X_i}) + F\left( {{x_i};q} \right)} \right)}$$
  4. *F*_*m*_(*x*) = *F*(*x*; *q*_*m*_)
  5. *T*_*m*_(*x*) = *T*_*m*−1_(*x*) + *ν*·*F*_*m*_(*x*)
  6. $${\text{write}}\left\{ {{F_m}(x)} \right\}_I^M$$

2. Choose coefficients $\left\{ c_m\right\} _{O}^{M}$ After all the base learners $\left\{ F_m(x) \right\} _{I}^{M}$ = $\left\{ F(x;q_m) \right\} _{I}^{M}$ have been selected the coefficients are obtained by linear regression: $\left\{ \hat{c_m} \right\} = arg min\sum _{I=1}^{N}L\left( {y_i,co+\sum _{m=1}^{M}cmF_m(x_i)}\right) \lambda \cdot Q(c)$, where $Q_c$ is the complexity penalty and $\lambda$ represents the meta-parameter. The other three parameters *L*, $\eta$ $\nu$, *L* represent the loss function, $\eta$ is responsible for data distribution and $S(\eta )$ represents a random sample that is the same size or less than the original data. If the values of $\eta$ are smaller the diversity of the ensemble will increase, also, $\eta$ influences computing time. $\nu$, regulates the alarms to the loss function.

The algorithm explains the start of an ensemble $T_0$ with a function (Line 1) which can be zero or any other constant. Then a leaner $F_m$ is included into the process. $T_m-1$ displays the ensemble of the base learners till $m-1$. $q_{m}= argmin_q\ldots$ finds the lowest error base leaner on a selected data set. That is a base learner is chosen that when combining with other selected learners best approximates the response. The new base leaner is then added to the ensemble which is represented by $F_m$. After *M* base learner have been created the algorithm ends the process.

Bagging: This is a combination method where each ensemble is trained using dissimilar training sets which are generated by sampling the original set, choosing *N* items uniformly at random with replacement [113]. The missing values predictions of the algorithms are then combined by averaging or voting. One major high notes of bagging it is that it is a standout and simple ensemble methods to implement and has great execution.

AdaBoost: Boosting is the procedure of taking weak learning missing handling algorithms and turning them into strong learning algorithms. Like bagging, boosting also re-samples data to create ensembles, which are then combined by majority voting. However, similarities end there. Different variations of Boosting have been done and proved to be good as far as expectation exactness in an assortment of uses. Its major drawback is the slow training speed of boosted trees [114]. However, the highlight of AdaBoost is that it can be utilised to enhance the performances of other data mining algorithms regardless of their nature [115].

Stacking: Stacking is a mechanism that combines different types of models that have been learned in the task into one. The missing value predictions of different models gives an input on a meta-level classifier and the output of this meta classifier will be the final prediction [116]. The major component in stacking is the optimal features and the algorithm for learning at the meta-level [117]. Instead of choosing one generalisation out of multiple generalisations, stacking combines them by using the output of base classifiers as inputs into the new space, stacking then makes predictions from new space. The stacking is also considered as an ensemble for further research in the context of base-level classifiers created by different learning algorithms [118].

Approaches in literature on missing values handling using ensemble methods are discussed in the following. Authors in Khan et al. [119], proposed a bootstrapping ensemble to model uncertainty and variety in the data that has high missingness. They performed an extensive evaluation of their approach by varying the missingness ratio of the missing data. Their results illustrated that bootstrapping is the most robust method for missing value imputation even at a high missingness ratio of up to 30 percent. However, for a small missingness ratio of 10 percent the ensemble strategy performed equivalently to other approaches but better than single imputation. Furthermore, the study was carried out using the MCAR missingness mechanism only, making their findings to be valid solely for this type of missingness.

Also, in another study Aleryani et al. [120] the authors proposed a Multiple Imputation Ensembles approach for handling with missing data in classification problems. They combined multiple imputation and ensemble techniques and compared two types of ensembles namely, bagging and stacking. The approach was termed robust as 20 benchmark datasets from the UCI machine learning repository were used. An increasing amount of missing data completely at random was simulated for all the data sets. It was reported that the approach performed well. However, it was not possible for the experiments results to be directly compared to other works on related work since different datasets and experimental set-ups were used.

Moreover, in Wang and Chen [121], a new approach for missing data using a three-way ensemble algorithm based on the imputation result was proposed. The objects with no missing values were firstly clustered by using a clustering method, then missing objects were filled using mean attributes of each cluster. The experimental results of the study on UCI machine learning repository data sets verify that the proposed algorithm was effective. However, like many other approaches in literature a missing value mechanism was not considered.

Also, in Madhu et al. [122], the researchers developed a novel ensemble imputation approach named the missXGBoost imputation technique. The technique has proven to be suitable for continuous attributes of medical applications. The missXGBoost method imputed plausible missing values in the original dataset and evaluated the classifier accuracy. The study experimental results demonstrated that the proposed imputation approach accuracy was better than the other traditional imputation methods. Furthermore, the method could be applied to high-dimensional mixed-type attributes data sets.

In another research a bagging and boosting ensemble algorithms as methods for handling missing data was proposed [123]. The proposed technique was compared with the existing methods by simulation and then applied to analyse a real large dataset to obtain realistic results. The researchers concluded that there is a lot of work to further experiment with their approach.

Table 1, presents a summary of different techniques in literature that used machine learning techniques to handle missing values. We present the general objective of the studies, the type of data set they used for their experiments, the missing mechanism followed and the limitations of the studies.

**Table 1:** A summary of various missing data techniques in machine learning

Refs.	DataSet	Performance objective	Mechanism	Summary	Limitations
[124]	Balance, Breast, Glass, Bupa, Cmc, Iris, Housing, Ionosphere, wine	To study the influence of noise on missing value handling methods when noise and missing values distributed throughout the dataset	MCAR, MAR, MNAR	The technique proved that noise had a negative effect on imputation methods, particularly when the noise level is high	Division of qualitative values may have been a problem
[85]	German, Glass(g2), heart-statlo, ionosphere, kr-vs-kp, labor, Pima-indians, sonar, balance-scale, iris, waveform, lymphography, vehicle, anneal, glass, satimage, image, zoo, LED, vowel, letter	Experimenting methods for handling incomplete training and test data for different missing data with various proportions and mechanisms	MCAR, MAR	In this technique an understanding of the relative strengths and weaknesses of decision trees for missing value imputation was discussed	The approach did not consider correlations between features
[125]	Los Angeles ozone pollution and Simulated data	To study classification and regression problems using a variety of missing data mechanisms in order to compare the approaches on high dimensional problems	MCAR, MAR	Here the authors tested the potential of imputation technique’s dependence on the correlation structure of the data	Random choice of missing values may have weakened the experiment consistency
[38]	Breast Cancer	To evaluate the performance of statistical and machine learning imputation techniques that were used to predict recurrence in breast cancer patient data		The machine learning techniques proved to be the most suited imputation and led to a significant enhancement of prognosis accuracy compared to statistical techniques	One type of data was used for the imputation model, therefore, the presented results may not generalise to different datasets
[126]	Iris, Wine, Voting, Tic-Tiac-Toe, Hepatitis	To propose a novel technique to impute missing values based on feature relevance	MCAR, MAR	The approach employed mutual information to measure feature relevance and proved to reduce classification bias	Random choice of missing values may have weakened the experiment consistency
[127]	Liver, Diabetis, Breast Cancer, Heart, WDSC, Sonar	Experimented on missing data handling using Random Forests and specifically analysed the impact of correlation of features on the imputation results	MCAR, MAR, MNAR	The imputation approach was reported to be generally robust with performance improving when increasing correlation	Random choice of missing values in MNAR could have weakened the consistency of the experiment
[128]	Wine , Simulated	To create an improved imputation algorithm for handling missing values	MCAR, MAR, MNAR	Demonstrated the superiority of a new algorithm to existing imputation methods on accuracy of imputing missing data	Features may have had different percentages of missing data, also MAR and MNAR may have been weakened
[129]	De novo simulation, Health surveys S1, S2 and S3	To compare various techniques of combining internal validation with multiple imputation	MCAR,MAR	The approach was regarded to be comprehensive with regard to the use of simulated and real data with different data characteristics, validation strategies and performance measures	The approach influenced potential bias by the relationship between effect strengths and missingness in covariates
[130]	Pima Indian Diabetes dataset	To experiment on missing values approach that takes into account feature relevance		The results of the technique proved that the hybrid algorithm was better than the existing methods in terms of accuracy, RMSE and MAE	Missing values mechanism was not considered
[13]	Iris, Voting, Hepatitis	Proposed an iterative KNN that took into account the presence of the class labels	MCAR, MAR	The technique considered class labels and proved to perform good against other imputation methods	The approach has not been theoretically proven to converge, though it was empirically shown
[74]	Camel, Ant, Ivy, Arc, Pcs, Mwl, KC3, Mc2	To develop a novel incomplete-instance based imputation approach that utilized cross-validation to improve the parameters for each missing value	MCAR, MAR	The study demonstrated that their approach was superior to other missing values approaches	
[131]	Blood, breast-cancer, ecoli, glass, ionosphere, iris, Magic, optdigits, pendigits, pima, segment, sonar, waveform, wine, yeast, balance-scale, Car, chess-c, chess-m, CNAE-9, lymphography, mushroom, nursery, promoters, SPECT, tic-tac-toe, abalone, acute, card, contraceptive, German, heart, liver, zoo	To develop a missing handling approach is introduced with effective imputation results	MCAR	The method was based on calculating the class center of every class and using the distances between it and the observed data to define a threshold for imputation. The method performed better and had less imputation time	Only one missing mechanism was implemented
[132]	Groundwater	Developed a multiple imputation method that can handle the missingness in ground water dataset with high rate of missing values	MAR	Here the technique used to handle the missing values, was chosen looking at its ability to consider the relationships between the variables of interest	There was no prior knowledge on the label of missing data which may have provided difficulty when performing imputation
[133]	Dukes’ B colon cancer, the Mice Protein Expression and Yeast	Developed a novel hybrid Fuzzy C means Rough parameter missing value imputation method		The technique handled the vagueness and coarseness in the dataset and proved to produce better imputation results	There was no report of missing values mechanisms used for the experiment
[134]	Forest fire, Glass, Housing, Iris, MPG, MV, Stocks, Wine	The method proposed a variant of the forward stage-wise regression algorithm for data imputation by modelling the missing values as random variables following a Gaussian mixture distribution. Categorical		The method proved to be effective compared to other approaches that combined standard missing data approaches and the original FSR algorithm	There was no report of missing values mechanisms used for the experiment
[135]	Weather dataset	This method applied four(Likewise, Multiple imputation, KNN, MICE) missing data handling methods to the training data before classification		Of the imputation methods applied the authors concluded that the most effective missing data imputation method for photovoltaic forecasting was the KNN method	There was no report of missing values mechanisms used for the experiment
[136]	Air quality data	To make time series prediction for missing values using three machine learning algorithms and identify the best method		The study concluded that deep learning performed better when data was large and machine learning models produced better results when the data was less	Heavy costs in time consumption and computational powers for training when implementing their most effective method (deep learning)
[137]	Traumatic Brain Injury and Diabetes	To demonstrate how performance varies with different missing value mechanisms and the imputation method used and further demonstrate how MNAR is an important tool to give confidence that valid results are obtained using multiple imputation and complete case analysis	MCAR, MAR, MNAR	The study showed that both complete case analysis and multiple imputation can produce unbiased results under more conditions	The method was limited by the absence of nonlinear terms in the substantive models
[138]	Grades Dataset	To develop a new decision tree approach for missing data handling	MCAR, MAR, MNAR	The method produced a higher accuracy compared to other missing values handling techniques and had more interpretable classifier	The algorithm suffered from a weakness when the gating variable had no predictive power
[139]	Air Pressure System data	The study proposed a sorted missing percentages approach for filtering attributes when building machine learning classification model using sensor readings with missing data		The technique proved to be effective for scenarios dealing with missing data in industrial sensor data analysis	The proposed approach could not meet the needs of automation
[139]	Abalone and Boston Housing	To experiment the reliability of missing value handling at not missing at random	MAR	The results of the study indicated that the approach achieved satisfactory performance in solving the lower incomplete problem compared to other six methods	The approach did not consider any missingness rate which may have affected the analysis
[140]	Cleveland Heart disease	Proposed a systematic methodology for the identification of missing values using the KNN, MICE, mean, and mode with four classifiers Naive Bayes, SVM, logistic regression, and random forest		The result of the study demonstrated that MICE imputation performed better than other imputation methods used on the study	The approach compared stage of the art methods with simple imputation methods, mean and mode that are bias and unrealistic results
[141]	Iris, Wine, Ecoli and Sonar datasets	To retrieve missing data by considering the attribute correlation in the imputation process using a class center-based adaptive approach using the firefly algorithm	MCAR	The result of the experiment demonstrated that the class center-based firefly algorithm was an efficient method for handling missing values	Imputation was tested on only one missing value mechanism
[15]	Abalone, Iris, Lymphography and Parkinsons	Proposed a novel tuple-based region splitting imputation approach that used a new metric, mean integrity rate to measure the missing degree of a dataset to impute various types missing data		The region splitting imputation model outperformed the competitive models of imputation	Random generator was used to impute missing values and other mechanisms for missing values were not considered
[142]	Artificial and real metabolomics data	To develop a new kernel weight function-based imputation approach that handles missing values and outliers	MAR	The proposed kernel weight-based approach proved to be superior compared to other data imputation techniques	The method was experimented on one type of dataset and may not perform as reported on other types of data

## Performance metrics for missing data imputation

The performance evaluation of different missing values approaches in machine learning problems can be done using different criteria, on this section we discuss the most used which are, Mean Absolute Error (MAE), Mean Squared Error (MSE), Root Mean Squared Error (RMSE) and Area under the curve (AUC).

### Mean Absolute Error (MAE)

MAE measures the average difference between imputed values and true values defined as:

18 $$\text{ MAE } = \frac{1}{m}\sum _{i=1}^{m}\left| y_i- \hat{y_i} \right|$$

### Mean Squared Error (MSE)

While MSE is equal to the sum of variance and squared predicted missing value as in the following equation:

19 $$\text{ MSE } = \frac{1}{m}\sum _{i=1}^{m} ( y_i- \bar{y_i} )^2$$

### Root Mean Square Error (RMSE)

RMSE computes the difference in imputed values and actual values as follows:

20 $$\text{ RMSE }=\sqrt{\frac{1}{m}\sum _{i-1}^{m}(y_{i}-{\bar{y}}_{{i}})^2}$$

MSE measures the average squared difference between the predicted missing values and the actual value, while RMSE represents the standard deviation of the differences between estimated missing values and observed values. Where *m* is the number of observations, $y_i$ is the observed values and ${\bar{y}}_{i}$ is the estimated missing value. A small value as an output for these performance metrics means that the estimated value is close to the real value.

### Area under the curve (AUC)

AUC is the representation of the degree or measure of separability and is used as a summary of the Root Receiver Operator Characteristic (ROC) curve, which is curve is a visualisation graph representing imputation performance [143]. The AUC is represented by the true positive rate (TPR) and the false positive rate (FPR). Where the TPR is the proportion of correctly imputed positives of all positives and the TPR is the proportion of all negatives that are wrongly imputed as positives [144]. The true positive rate and the false positive rate are defined as:

21 $$TPR= \frac{TP}{TP+FN}$$

22 $$FPR= \frac{FP}{FP+TN}$$

The major advantages of the MSE and RMSE is that they provide a quadratic loss function. Also, uncertainty in forecasting is measured when they are used. However, MSE and RMSE are highly influenced by extreme values [145]. While MAE is not influenced by extreme values, also a more natural measure and unambiguous [146]. Most studies in research are found to mostly use the RMSE for missing value imputation evaluation [147–149]. Although some studies have proposed valid evidence against the use of RMSE in favour of MAE due to its less sensitive to extreme values [150]. The authors further advised against the reporting of RMSE in literature and strongly recommended the use of MAE [146, 150]. However, Chai and Draxler [145] partially disputed the conclusions and introduced arguments against avoiding RMSE. They contended that RMSE was appropriate to represent model performance than the MAE. The AUC like other performance measures also has its advantages, it allows for a visualised graphical representation of imputation performance and is also unaffected by abnormal distributions in the population and decision criterion [151]. However, actual decision thresholds are usually not represented by AUC graph and it overlooks the probability of predicted values and the goodness-of-fit of the model [152]. Discussions on which metric to use in literature have proven that performance measures are not equivalent to each other, and one cannot easily derive the value of one from another. Nonetheless, all distance measurements (MSE, RMSE, MAE and AUC) help to quantify the accuracy of the estimated solution compared to the actual non-missing data and an appropriate method must be selected for the most appropriate analysis for the question being addressed.

## Comparisons

In this section, we discuss observations made, and present a comparative analysis on performance matrices, publications made and the year of publication for different research.

### Evaluation metrics

Table 2 shows details of different selected articles that were researched on missing data handling using different techniques and the journals, books, conference they were published on. We selected articles in Table 3 for metrics used to evaluate different missing values handling approaches. The selection is based on whether the article covers the most popular evaluation methods.

**Table 2:** Details of selected articles for missing values handling

Citation	Year	Publisher	Article	Journal/conference/book chapter
[153]	2020	Applied Science	Missing value imputation in stature estimation by learning algorithms using anthropometric data: a comparative study	Multidisciplinary Digital Publishing Institute
[139]	2020	Applied Science	Evaluating machine learning classification using sorted missing percentage technique based on missing data	Multidisciplinary Digital Publishing Institute
[154]	2020	Biometrical Journal	Multiple imputation methods for handling missing values in longitudinal studies with sampling weights: comparison of methods implemented in Stata	Wiley Online Library
[155]	2019	Applied Artificial Intelligence	Comparison of performance of data imputation methods for numeric dataset	Taylor and Francis
[8]	2006	Elsevier	A gentle introduction to imputation of missing values	Journal of clinical epidemiology
[127]	2017	Elsevier	Adjusted weight voting algorithm for random forests in handling missing values	Pattern Recognition
[60]	2017	Elsevier	kNN-IS: an Iterative Spark-based design of the k-Nearest Neighbors classifier for big data	Knowledge-Based Systems
[156]	2021	Elsevier	Ground PM2. 5 prediction using imputed MAIAC AOD with uncertainty quantification	Environmental Pollution
[157]	2021	Elsevier	A neural network approach for traffic prediction and routing with missing data imputation for intelligent transportation system	Expert Systems with Applications
[158]	2021	Elsevier	Handling complex missing data using random forest approach for an air quality monitoring dataset: a case study of Kuwait environmental data (2012 to 2018)	Multidisciplinary Digital Publishing Institute
[159]	2021	Elsevier	HA new method of data missing estimation with FNN-based tensor heterogeneous ensemble learning for internet of vehicle	Neurocomputing
[111]	2006	IEEE	Ensemble based systems in decision making	IEEE Circuits and systems magazine
[160]	2010	IEEE	Missing value estimation for mixed-attribute data sets	IEEE Transactions on Knowledge and Data Engineering
[161]	2014	IEEE	Modeling and optimization for big data analytics:(statistical) learning tools for our era of data deluge	IEEE Signal Processing Magazine
[2]	2014	IEEE	Handling missing data problems with sampling methods	2014 International Conference on Advanced Networking Distributed Systems and Applications
[123]	2018	IEEE	An imputation method for missing data based on an extreme learning machine auto-encoder	IEEE ACCESS
[162]	2018	IEEE	A data imputation model in phasor measurement units based on bagged averaging of multiple linear regression	IEEE ACCESS
[163]	2018	IEEE	Missing network data a comparison of different imputation methods	2018 IEEE/ACM International Conference on Advances in Social Networks Analysis and Mining (ASONAM)
[164]	2018	IEEE	MIAEC: missing data imputation based on the evidence chain	IEEE ACCESS
[165]	2018	IEEE	A survey on data imputation techniques: water distribution system as a use case	IEEE ACCESS
[166]	2019	IEEE	Missing values estimation on multivariate dataset: comparison of three type methods approach	International Conference on Information and Communications Technology (ICOIACT)
[122]	2019	IEEE	A novel algorithm for missing data imputation on machine learning	2019 International Conference on Smart Systems and Inventive Technology (ICSSIT)
[167]	2020	IEEE	Approaches to dealing with missing data in railway asset management	IEEE ACCESS
[168]	2020	IEEE	Traffic data imputation and prediction: an efficient realization of deep learning	IEEE ACCESS
[169]	2020	IEEE	Iterative robust semi-supervised missing data imputation	IEEE ACCESS
[170]	2021	IEEE	Missing network data a comparison of different imputation methods Neighborhood-aware autoencoder for missing value imputation	2020 28th European Signal Processing Conference (EUSIPCO)
[171]	2021	IEEE	Hybrid missing value imputation algorithms using fuzzy C-means and vaguely quantified rough set	IEEE Transactions on Fuzzy Systems
[56]	2016	SAGE Publications	Multiple imputation in the presence of high-dimensional data	Statistical Methods in Medical Research
[172]	2020	Sensors	A method for sensor-based activity recognition in missing data scenario	Multidisciplinary Digital Publishing Institute
[31]	2012	Springer	Analysis of missing data	Missing data
[65]	2015	Springer	CKNNI: an improved knn-based missing value handling technique	International Conference on Intelligent Computing
[126]	2015	Springer	Missing data imputation by K nearest neighbours based on grey relational structure and mutual information	Applied Intelligence
[63]	2016	Springer	Nearest neighbor imputation algorithms: a critical evaluation	BMC medical informatics and decision making
[105]	2017	Springer	Multiple imputation and ensemble learning for classification with incomplete data	Intelligent and Evolutionary Systems
[68]	2018	Springer	NS-kNN: a modified k-nearest neighbors approach for imputing metabolomics data	Metabolomics
[136]	2019	Springer	Analysis of interpolation algorithms for the missing values in IoT time series: a case of air quality in Taiwan	The Journal of Super computing
[39]	2020	Springer Open	SICE: an improved missing data imputation technique	Journal of Big Data
[138]	2020	Springer	BEST: a decision tree algorithm that handles missing values	Computational Statistics
[173]	2020	Springer	A new multi-view learning machine with incomplete data	Pattern Analysis and Applications
[140]	2021	Springer	Multistage model for accurate prediction of missing values using imputation methods in heart disease dataset	Innovative Data Communication Technologies and Application
[14]	2021	Springer	A new imputation method based on genetic programming and weighted KNN for symbolic regression with incomplete data	Soft Computing
[174]	2021	Springer	An exploration of online missing value imputation in non-stationary data stream	SN Computer Science
[175]	2021	Springer	Data imputation in wireless sensor network using deep learning techniques	Data Analytics and Management
[176]	2020	Sustainable and Resilient Infrastructure	Handling incomplete and missing data in water network database using imputation methods	Taylor and Francis

**Table 3:** Qualitative comparison between different missing data techniques in machine learning based on the performance metrics adopted

Publication	Performance metrics			
	RMSE	MAE	MSE	AUC
[125]	${\times }$	${\times }$	$\checkmark$	${\times }$
[129]	${\times }$	${\times }$	$\checkmark$	$\checkmark$
[74]	$\checkmark$	${\times }$	${\times }$	${\times }$
[127]	${\times }$	${\times }$	${\times }$	$\checkmark$
[131]	$\checkmark$	$\checkmark$	${\times }$	${\times }$
[133]	$\checkmark$	${\times }$	${\times }$	${\times }$
[135]	$\checkmark$	${\times }$	${\times }$	${\times }$
[136]	${\times }$	$\checkmark$	$\checkmark$	${\times }$
[126]	$\checkmark$	${\times }$	${\times }$	${\times }$
[130]	$\checkmark$	$\checkmark$	${\times }$	${\times }$
[128]	$\checkmark$	${\times }$	${\times }$	${\times }$
[139]	${\times }$	${\times }$	${\times }$	$\checkmark$
[138]	${\times }$	${\times }$	${\times }$	$\checkmark$
[140]	$\checkmark$	${\times }$	${\times }$	${\times }$
[174]	$\checkmark$	${\times }$	${\times }$	${\times }$
[156]	$\checkmark$	${\times }$	${\times }$	${\times }$
[158]	$\checkmark$	$\checkmark$	${\times }$	${\times }$
[170]	$\checkmark$	${\times }$	${\times }$	${\times }$
[15]	$\checkmark$	${\times }$	${\times }$	${\times }$
[142]	${\times }$	${\times }$	$\checkmark$	$\checkmark$
[38]	${\times }$	${\times }$	$\checkmark$	$\checkmark$
[13]	$\checkmark$	${\times }$	${\times }$	${\times }$
[141]	$\checkmark$	${\times }$	${\times }$	${\times }$

### Experimental evaluation on machine learning methods

An experimental evaluation on two of the most representative machine learning techniques on two datasets was done to show experimental results. Considering the possible variability on performances of algorithms, the experiment was done on more than one algorithm based on the Iris and ID fan datasets. The Iris dataset is a very popular dataset which was originally published at UCI Machine Learning Repository introduced by Fisher [177], for an application of discriminant analysis for three species of Iris flowers (setosa, versicolor, and virginica), having four variables being length and width of the sepal and the petal (in cm). We also experimented on an Induced draft fan (ID fan) dataset from a local coal-fired power plant where real data of a coal power plant fan system was recorded. The dataset contains readings for the month of February 2021 of a single unit of the power plant. The ID fan vibrations are measured by sensors and were recorded by the technicians every 4 h when the plant was running. These variables specifically consist of bearing vibrations and temperatures, at the fan non-drive end (FNDE) and fan drive end (FDE), motor temperatures and vibrations, at the motor non-drive end (MNDE) and motor drive end (MDE). The values of the ID fan are recorded as part of the daily power plant monitoring system. Both the Iris and ID Fan datasets contain 150 instances with no missing values. Our method simulates the missing values on sepal length and petal width of the Iris data and the Vibrations on the ID fan data. The target missingness fraction was set to MCAR by setting the probability of a value to being missing to 5%, 10% and 15% across all observations. RMSE performance measure was then used to help quantify the accuracy of the estimated values compared to the actual non-missing data.

After simulation of missing values, KNN imputation was implemented to replace the missing values. Firstly, when implementing the imputation method, the nearest neighbors (K) must be chosen. The value of K was chosen based on experimental results starting with K = 1 and stopped at K = 5, the best accurate estimation value of K was then used for the experiment which was K = 4. The Euclidean distance measure was used on the KNN imputation algorithm. The RF missForest algorithm was then implemented, which is a nonparametric imputation method based on the random forest. For every variable missForest fits a random forest on the observed values and then predicts the missing variables. The process of training and predicting of missForest is repeated in an iterative process until a number of iterations are reached. The missForest ran for three iterations and stopped. The iterative stopping criterion was reached when the difference between the previously imputed values and the newly imputed data increased for the first time with respect to both variable types. Multiple iterations enabled the algorithm to be trained on better quality data that it previously predicted.

**Fig. 2:**
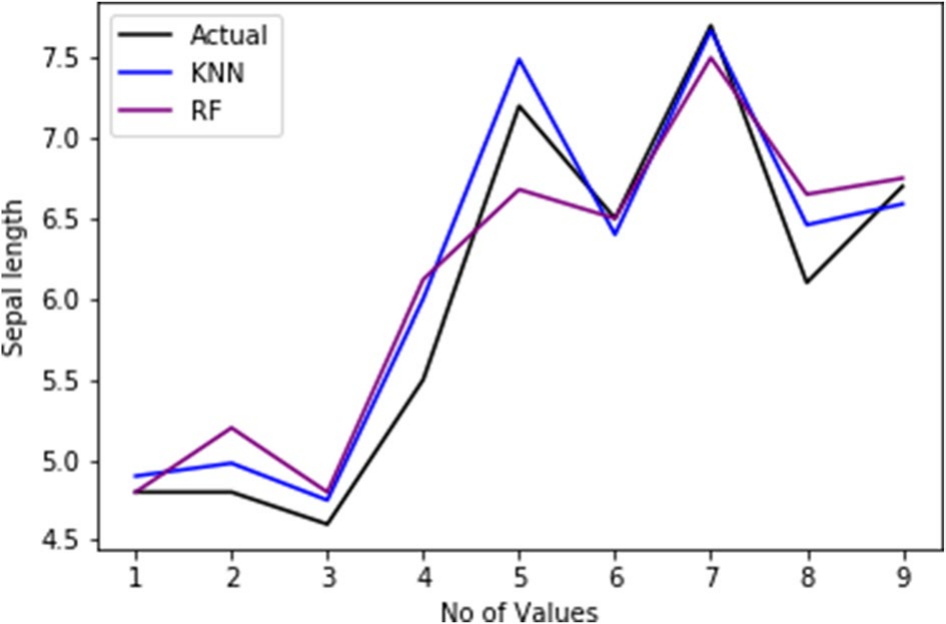
Comparison of KNN and RF Imputed values with the actual values on Sepal Length at 15% ratio

**Fig. 3:**
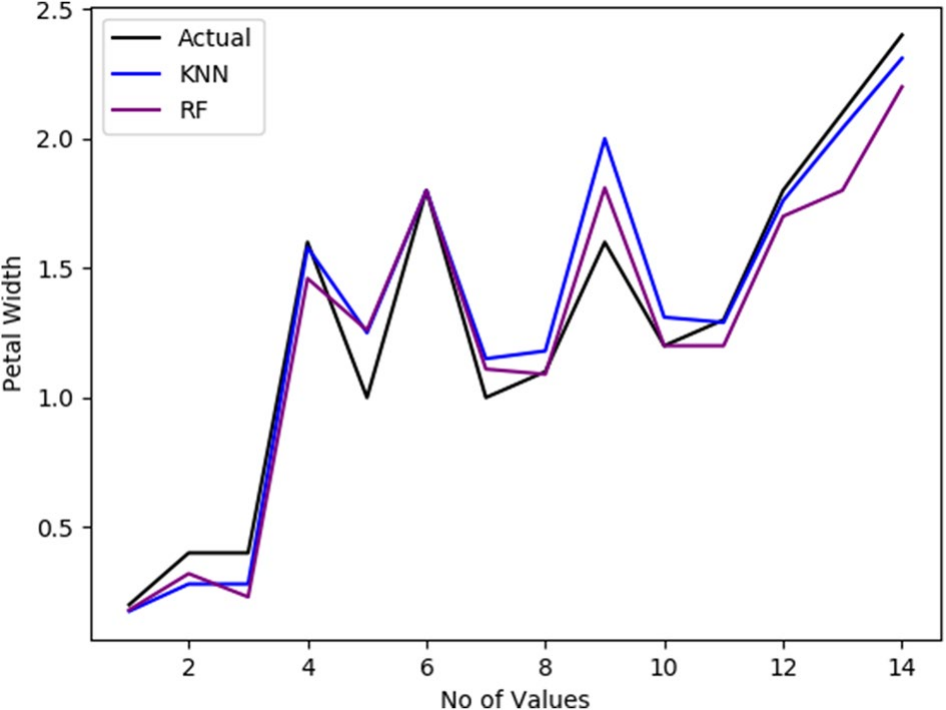
Comparison of KNN and RF imputed values with the actual values on Petal width at 15%

**Fig. 4:**
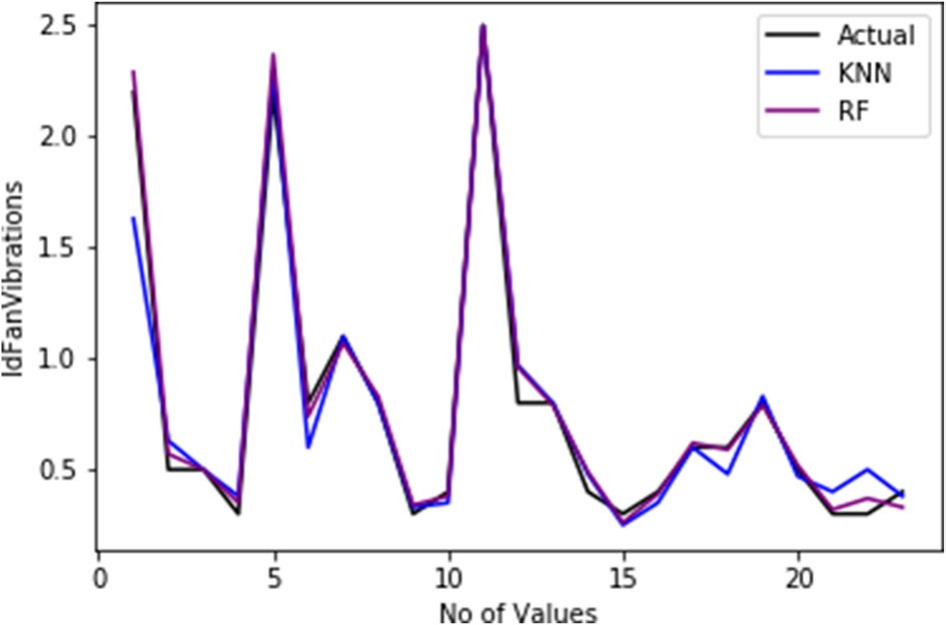
Comparison of KNN and RF imputed and actual values at 15% on the Id fan dataset

We present the different performances of the KNN and RF algorithms on imputed values with the actual values at a missing rate of 15% on Figs. 2 and 3 on the Iris dataset. Table 4 represents the RMSE of the KNN and RF algorithms at different imputation ratios on the Iris data set. The experiment demonstrated that the KNN imputation performed better than the RF imputation on the Iris dataset at 10% and 15% missingness ratios. While the RF performed better than the KNN at 5% on the Iris dataset. While, Fig. 4 shows the comparison of KNN and RF algorithms on imputed and actual values at a missing rate of 15% on the ID fan dataset. Table 5 shows that the RF performed better than the KNN on the ID fan data sets at all missing value percentages.

**Table 4:** RMSE of KNN and RF imputation at different ratios on the Iris dataset

Missing ratio%	KNN	RF
5	0.6693	0.6486
10	0.2382	0.2860
15	0.1932	0.2578

**Table 5:** RMSE of KNN and RF imputation at different ratios on the ID fan dataset

Missing ratio%	KNN	RF
5	0.2099	0.0549
10	0.1581	0.0416
15	0.1487	0.0654

## Conclusion and future work

This paper provides a comprehensive review on the problem of missing values, including missing data mechanisms, missingness types and a considerable number of missing data handling approaches, for different applications and scenarios. It also provides reference for researchers on choosing an appropriate method for handling missing values. Also, an imputation experiment was done on the KNN and RF algorithms for imputation on the Iris and novel ID fan datasets to demonstrate how popular imputation algorithms perform. KNN imputation performed better than the RF imputation using RMSE as an evaluation measure on the Iris data on two missingness ratios and the RF performed better than the KNN on the ID fan data on all missingness ratios. This has led to a conclusion that, the precision and accuracy of machine learning imputation algorithms depend strongly on the type of data being analysed, and that there is no clear indication that favours one method over the other. The review demonstrated existence of many limitations on existing missing vales methods. It was notable that RSME is mostly used as an evaluation metrics and metrics are not mostly used together, which is one of the main limitations of current literature and should be considered in future research. Also, most reviewed works show different domain datasets that are not as big as real world datasets, which often contain a very large number of diverse features. Therefore, further work is needed to explore the possibilities of new methods of handling missing data in real world big data.

## Data Availability

The availability one data sources is explained in the manuscript with a reference.

## Appendix

The table explains some notations used on the manuscript. Table 6 illustrates the summary of the notations and definitions used on the paper.

**Table 6:** Summary of notation and definitions

Notation	Definition
*b*	The Bias
$Dist_{xy}$	The Euclidean distance
$f(y_{obs})$	The complete data in the data set
*H*	The separating hyper-plane
*k*	The data attributes
*m*	The number of observations
*n*	The number of observed data
*p*	The probability of missing data
*q*	The vector indicating the missingness association
*R*	The missing value matrix
$v_j$	Attributes containing missing data
*w*	The weight vector
$w_j$	Nearest neighbours
*x*	The input vector
$x_i\cdot$	The error terms for un-predicted determinants of ${\bar{y}}$
$X_k$	Mean estimation
*Y*	The matrix of an entire data set
$Y_m$	The missing data in *R*
$Y_o$	The observed data in *R*
${\bar{y}}$	The predicted data

## Abbreviations

AUC: Area under the curve
CART: Classification and Regression Trees
FNDE: Fan non-drive end
FDE: Fan drive end
MEA: Mean Absolute Error
MDE: Motor drive end
MSE: Mean Squared Error
MNDE: Motor non-drive end
KNN: K nearest neighbor
MAR: Missing at random
MCAR: Missing completely at random
MNAR: Missing not at random
RF: Random Forests
RMSE: Root Mean Squared Error
UCI: University of California
SVM: Support vector machines
